# Does the association of education with breast cancer replicate within twin pairs? A register-based study on Danish female twins

**DOI:** 10.1038/sj.bjc.6606090

**Published:** 2011-02-01

**Authors:** M Madsen, P K Andersen, M Gerster, A-M Nybo Andersen, K Christensen, M Osler

**Affiliations:** 1 Unit of Epidemiology, University of Southern Denmark, The Danish Twin Registry, DARC, University of Southern Denmark, J.B. Winsløwsvej 9B, DK-5000 Odense C, Denmark; 2 Institute of Public Health, Division of Biostatistics, University of Copenhagen, Copenhagen, Denmark; 3 Unit of Biostatistics, University of Southern Denmark, Odense, Denmark; 4 Institute of Public Health, Division of Social Medicine, Copenhagen, Denmark; 5 Research Centre for Prevention and Health, Glostrup University Hospital, Glostrup, Denmark; 6 Danish Aging Research Centre, Department of Epidemiology, University of Southern Denmark, J. B. Winslowsvej 9, DK-5000 Odense C, Denmark

**Keywords:** twin study, education, social class, breast neoplasm

## Abstract

**Background:**

A positive association between socio-economic position and breast cancer has been widely observed, but not hitherto within twin pairs, where shared familial factors were adjusted for.

**Methods:**

We used data on education and other factors from the Danish Twin Registry, The Danish Cancer Registry, and official registers on a total of 16 310 twins. Unpaired and intrapair Cox regression analyses were compared.

**Results:**

In the unpaired analysis, an educational gradient in breast cancer risk was found. Similar results were seen in the intrapair analyses of all twins, although no longer statistically significant. When intrapair analyses were stratified on zygosity, the effect of education was attenuated in the monozygotic twins.

**Conclusion:**

The main findings support an effect of education beyond shared familial factors.

In Denmark, breast cancer (BC) incidence has increased during recent decades, and it is now the most frequent cancer in women (Ferlay *et al*, 2007). In contrast to most health outcomes, a positive social gradient has been observed in BC, showing a higher risk in higher socio-economic position (SEP) groups (Kongevinas *et al*, 1997).

Several BC risk factors are well established: high age at first birth (AFB), low parity (Ma *et al*, 2006), hormonal replacement therapy (Porch *et al*, 2002; Tjonneland *et al*, 2004), high body mass index (van den Brandt *et al*, 2000; Ahlgren *et al*, 2004), and high alcohol consumption (Boyle and Boffetta, 2009). In recent years, there has also been interest in *in utero*- and early-life factors, including high birth weight (Silva *et al*, 2008; Oberg *et al*, 2009). Moreover, evidence suggests that child growth and childhood diet may convey the risk (Frazier *et al*, 2003; Ahlgren *et al* 2004; De Stavola *et al*, 2004; Michels *et al*, 2006). The BC aetiology seems to be influenced by different exposures that exert their influence throughout the life course (Lynch and Smith, 2005). Although modest, a genetic component of BC has also been demonstrated. In a large Nordic twin study, non-shared environment was found to influence the risk of BC the most (explaining 67% of the variance), while shared environmental factors seemed to have a negligible role (Lichtenstein *et al*, 2000). This may suggest that adult factors have a much larger effect than early-life factors. However, the power to disentangle genetic and shared environmental effects in heritability studies is limited.

The observed social differentials in BC may be driven by socially patterned adult factors such as AFB and parity, as better-educated women are more likely to postpone their pregnancies and have fewer children. Alternatively, the differentials may be a result of selection processes in which underlying factors, such as genes or childhood environment, influence SEP and the risk of BC, thus creating a spurious association (Smith *et al*, 1994). Previous studies on SEP and cancer have mostly dealt with mortality as opposed to incidence, which may complicate the picture if the social patterning of incidence and survival from cancer differs. However, a recent study found no effect of SEP in childhood on BC incidence when adult SEP was adjusted for (de Kok *et al*, 2008).

Twins provide a unique model to disentangle contributions from adult, childhood, and genetic factors as twins share their intrauterine- and early environment and are partly (dizygotic (DZ) twins) or fully (monozygotic (MZ) twins) matched on genetic setup. We aimed, by using education as an indicator of SEP, to separate the effect of SEP on BC from that of shared familial factors.

## Materials and methods

In the Danish Twin Registry, all female twins born between 1921 and 1974 were considered for the study. After restrictions to sex-liked intact twin pairs of known zygosity, alive, and resident in Denmark at the beginning of follow-up (1 January 1980 or the date the participants turned 30 years if younger than 30 years in 1980) with no previous BC diagnosis and with full information on education, the final study population consisted of 16 310 twins, including 6268 MZ and 10 042 dizygotic same sex twins (DZSS).

Information on education came from Statistics Denmark and was operationalised as educational status according to the International Standard Classification of Education (Institute For Statistics, 2006) and length of education. Educational status was categorised into primary/lower secondary (primary) and upper secondary/tertiary education (secondary/tertiary) while length of education was included as a quantitative variable measured in years.

As parity and AFB are known to be strong determinants of BC and strongly correlated with education, we included these variables in a subanalysis to investigate the degree to which these factors might be driving the association between education and BC. Parity was constructed as a time-dependent variable (0, 1, 2+ births), while AFB was categorised as follows: no children, <26 years, 26–30 years, 31–35 years, >35 years. Owing to collinearity between the two variables, they were included in the model as a combined variable.

The Danish Cancer Registry contains information on all primary incident cancers in Denmark since 1943, except non-melanoma skin cancer. Reporting is mandatory and registered information comes from a cross-checking of different sources. Cases of BC were indentified through ICD-7 (1980–1993) and ICD-10 (1994–2006) codes (170 and C50, respectively).

### Data analysis

Using Cox regression, person years of follow-up were accumulated from baseline (age at 1 January 1980 or age 30 years) to the age at the end of follow-up (age at 31 December 2006), diagnosis of BC, death, or emigration, whichever was earliest. The analyses were adjusted for calendar time. Unpaired analyses were compared with intrapair analyses. The intrapair analyses were carried out by stratifying on ‘pair-number’ (Holt and Prentice, 1974), which permits the baseline hazard to vary freely between pairs while at the same time fixing it within pairs. The hazard function for twin *j* in pair *i* is given by *h*_*ij*_(*t*,*z*)=*h*_0*i*_(*t*) exp(*βz*_*ij*_), where *h*_0*i*_(*t*) is the pair-specific baseline hazard and *β* is the effect of education that applies to all subjects.

Differences in genetic relatedness between MZ (genetically identical) and DZ twins (share on average 50% of segregating genes) could further be exploited to make inferences about genetic and environmental confounding, respectively; assuming an effect of education in the unpaired analyses, genetic confounding would be indicated if the intrapair analyses showed a partial attenuation of the association in DZSS twins and a full attenuation in the MZ twins. Similarly, confounding from shared environment would be likely to account for the association if a full attenuation were observed in both DZSS and MZ twins. Finally, persistence of the association would be expected if education had an independent effect on BC beyond the effect of shared familial factors. Hazard ratios (HRs) were calculated and 95% confidence limits were corrected for interdependence of observations within twin pairs. In the intrapair analyses, a strata option was included (StataCorp. 2009, Stata Statistical Software: Release 11, College Station, TX, USA and StataCorp LP, College Station, TX, USA). Potential interaction between education and zygosity was examined.

## Results

In the cohort of 16 310 female twins, 518 developed BC during the follow-up period 1980–2006. As expected, more DZSS than MZ twins were discordant on education (Table 1). More twins were discordant by the quantitative definition, which expresses the difference in years of education within a twin pair. A larger mean difference in length of education was also observed among the DZSS twins. Additionally, a higher proportion of DZSS (44%) twins had a primary education compared with MZ twins (36%). The unpaired analysis of risk according to educational status (Table 2) shows a statistically significant increased risk associated with a secondary/tertiary education compared with primary education (HR=1.39, 95% confidence interval (CI)=1.15–1.67) for all twins. Similar results were seen when DZSS and MZ twins were analysed separately, and an analysis of potential interaction showed no evidence of modification of educational effect by zygosity (*P*=0.66). For the length of education, we observed a 4% increased risk of BC associated with one additional year of education (HR=1.04, 95% CI=1.01–1.07). The same pattern was seen when stratified on zygosity. The intrapair analysis of all twins showed an association with educational status of the same magnitude as in the unpaired analyses (HR=1.38), although no longer statistically significant (95% CI=0.95–2.01). When the analysis was split on zygosity, the association was attenuated in the MZ twins (HR=0.82, 95% CI=0.41–1.67). However, CIs were wide due to the limited number of events. For DZSS twins, the association persisted (HR=1.70, 95% CI=1.08–2.69). Similar trends were suggested in the analyses by length of education. No statistically significant interaction between education and zygosity was found in the intrapair analysis (*P*=0.35). When the analysis was adjusted for parity and AFB, the effect of education changed only negligibly (educational status: HR=1.37, 95% CI=1.14–1.65 and educational length: HR=1.04, 95% CI=1.01–1.07) and the effect of the combined covariate was statistically insignificant (*P*=0.61).

## Discussion

In this study, we examined the association between education and BC risk in Danish female twins with and without adjusting for familial background factors.

In the unpaired analyses we found the expected increased risk associated with a secondary/tertiary compared with a primary educational status of similar magnitude to that previously reported (Kongevinas *et al*, 1997; Carlsen *et al*, 2008). This association was replicated in the intrapair analyses for all twins, suggesting that the effect of education is not due to confounding from familial factors. This accords with the Nordic twin study (Lichtenstein *et al*, 2000) that suggests that non-shared factors are most important risk factors for BC as in the study that showed no effect of childhood SEP on incidence, when adult factors were adjusted for (de Kok *et al*, 2008).

When MZ twins were analysed separately, the association was completely attenuated. However, as only few MZ twin pairs were available for the intrapair analysis, this is likely a chance finding. If not, the results are suggestive of underlying genetic factors associated with both a high education and a high risk of BC. The fact that no partial attenuation was observed in DZSS twins, contrary to expectation, might be explained by the presence of complex gene–gene interactions where several genes need to be present simultaneously in order for a phenotype to occur. Alternatively, if MZ twins also share their environment more closely than DZSS twins (Horwitz *et al*, 2003), for example fertility patterns, this could account for the observed differences in attenuation of effect between MZ and DZSS twins. However, this was not supported in our data set where parity and AFB did not have a statistically significant impact on the risk of BC.

This study is the first to use the discordant twin-pair design to examine social inequality in BC. Several previous studies have used this approach to address causality in the social patterning of health and disease, but the population size available for this study allowed us to investigate a hard end point such as BC, which presents an interesting hypothesis because of its curious positive association with SEP. The prospective design and almost complete information in the registers ensure valid information about educational status and BC in this largely unselected twin population.

A key assumption in the discordant twin-pair logic is that twins who have grown up together are matched on early environment. Survey data indicate that this was the case for about 98% of the population (Madsen *et al*, 2010), so we do not expect this to be an important source of bias. Selection is another relevant issue. Twins who are discordant on education may comprise a selected group as they have obtained different educational statuses in spite of their common background and may not be representative of the twin population at large. Nevertheless, the discordant twin-pair method offers an exceptional opportunity to enhance validity in non-experimental designs.

In conclusion, the finding of an effect of education in the combined intrapair analysis suggests that education has an effect on BC risk beyond shared familial factors; parity and AFB did not seem to be driving this association. The finding of an attenuation of effect in MZ twins, suggesting genetic confounding, may serve as a hypothesis for testing in larger data sets.

## Figures and Tables

**Table 1:** Descriptives on educational status and length of education (mean number of years) according to zygosity in a population of Danish female twins (*N*=16 310)

	***N***	**%**	**Number of years**			
			**Mean**	**s.d.**	**Min**	**Max**
*DZSS (*N*=10 042)*						
*Educational status* a						
Primary	4395	44	8.0	1.3	7	10
Secondary/tertiary	5647	56	13.6	1.5	10.5	20
						
*Dicordance on education*						
Binary definition^b^	2706	27	4.7	1.7	0.5	10
Continuous definition^c^	6114	61	3.1	2.0	0.1	10
						
*MZ (*N*=6268)*						
*Educational status* a						
Primary	2269	36	8.2	1.3	6	10
Secondary/tertiary	3999	64	13.7	1.6	10.5	20
						
*Dicordance on education*						
Binary definition^b^	1174	19	4.2	1.7	0.5	11
Continuous definition^c^	3116	50	2.7	1.8	0.1	11

Abbreviations: DZSS=dizygotic same sex twins; MZ=Monozygotic twins.

a Measured according to ISCED (International Standard Classification of Education).

b Education discordant twins where one twin has a primary education and the co-twin has a secondary/tertiary education.

c Education discordant twins that differ in their length of education measured in years, that is, >0 years difference.

**Table 2:** Hazard ratios of breast cancer (1980–2006) according to educational status and length of education in a population of Danish female twins (*N*=16 310) showing results from Cox regression analyses stratified on zygosity

	**Unpaired analysis** a									**Intrapair analysis^b^**								
	**ALL^c^ *N*=16 310**			**DZSS *N*=10 042**			**MZ *N*=6268**			**ALL^c^ *N*=16 310**			**DZSS *N*=10 042**			**MZ *N*=6 268**		
	**HR^d^**	**CI 95%**	**Events**	**HR^d^**	**CI 95%**	**Events**	**HR^d^**	**CI 95%**	**Events**	**HR**	**CI 95%**	**Events**	**HR**	**CI 95%**	**Events**	**HR**	**CI 95%**	**Events**
			*518*			*344*			*174*			*127*			*91*			*36*
*Educational status* e																		
Primary	1	ref		1	ref		1	ref		1	ref		1	ref		1	ref	
Secondary/tertiary	**1.39**	1.15–1.67		**1.37**	1.09–1.71		**1.43**	1.04–1.97		1.38	0.95–2.01		**1.70**	1.08–2.69		0.82	0.41–1.67	
																		
												*263*			*183*			*80*
*Length of education* f																		
Number of years^g^	**1.04**	1.01–1.07		1.03	1.00–1.07		**1.07**	1.02–1.12		1.04	0.97–1.11		1.06	0.98–1.14		0.99	0.88–1.11	

Abbreviations: DZSS=dizygotic same sex twins; MZ=Monozygotic twins; CI=confidence interval; HR=hazard ratio. Bold numbers indicate statistical significance at 5% level.

a Unpaired analysis treating twins as individuals taking interdependence of observations into account by including a cluster term. The interpretation of HR is the risk of breast cancer in an individual with a secondary or tertiary education compared to a random individual with a primary education.

b Intrapair analysis of twins by inclusion of a strata statement. The interpretation of HR is the risk of breast cancer in a twin with a secondary or tertiary education compared to its co-twin with a primary education.

c ALL=monozygotic+dizygotic twins.

d Adjusted for calender time.

e Measured according to ISCED (International Standard Classification of Education).

f Measured as standard number years required to complete a given education.

g Quantitive variable. Interpretation of HR is the increase in risk of breast cancer associated with one additional year of education
